# 756. *Clostridioides difficile* Burden of Disease: A Prospective Population-Based Surveillance Study of Hospitalized Adults in Louisville, Kentucky

**DOI:** 10.1093/ofid/ofab466.953

**Published:** 2021-12-04

**Authors:** Stephen Furmanek, Ruth Carrico, Fredrick J Angulo, Joann Zamparo, Elisa Gonzalez, Kimbal D Ford, Julio Ramirez

**Affiliations:** 1 University of Louisville, School of Medicine, Division of Infectious Diseases, Louisville, KY; 2 University of Louisville, Louisville, Kentucky; 3 Pfizer, Inc., Collegeville, Pennsylvania

## Abstract

**Background:**

*Clostridioides difficile* (*C. difficile*) is an important cause of morbidity and mortality. *C. difficile* infection (CDI) may be frequently under-diagnosed because laboratory confirmation requires collection of a stool specimen from a patient with diarrhea and appropriate laboratory testing.

**Methods:**

A prospective population-based CDI surveillance study was launched in 8 adult hospitals in Louisville, Kentucky on September 16, 2019. Surveillance officers in each hospital identified all cases of new-onset diarrhea (≥3 loose stools in the past but not preceding 24 hours) in Louisville residents ≥50 years of age. After informed consent, stool samples were collected and tested at the University of Louisville reference laboratory for 1) glutamate dehydrogenase (GDH) and 2) *Clostridioides difficile* toxins A and B using C. DIFF QUIK CHEK COMPLETE®, Techlab. We defined CDI as GDH positive and toxin positive. The study was paused on April 3, 2020, due to COVID-19 restrictions.

**Results:**

There were 85,719 eligible patient-days during the study period. A total of 1541 patients had new-onset diarrhea corresponding to 1.8 cases of new-onset diarrhea per 100 eligible patient-days. We enrolled 84% (1291/1541) of patients with new-onset diarrhea and tested stool samples for *C. difficile* from 82% (1055/1291) for a testing density of 123 per 10,000 patient-days. Of the 1055 tested stool specimens, 73 (7%) were GDH positive and toxin positive (Figure 1) yielding a hospital-based CDI incidence of 8.5 CDI cases per 10,000 patient-days.

Figure 1. Patient Ascertainment Flow Chart

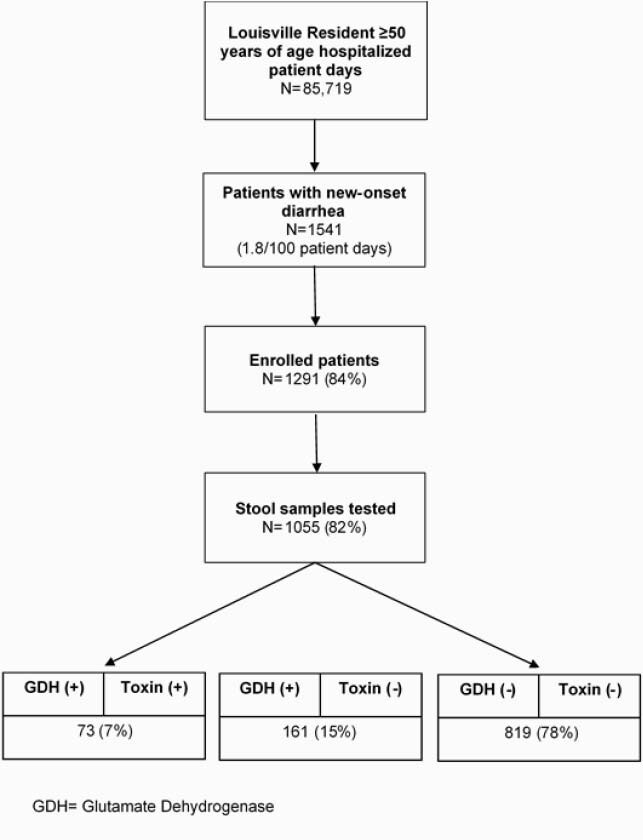

**Conclusion:**

New-onset diarrhea was common among hospitalized adults ≥50 years of age. CDI was frequently identified through stool specimens collected from eligible inpatients with new-onset diarrhea. Further analysis of these data and additional laboratory testing will contribute to a better understanding of the frequency of CDI underdiagnosis and the burden of CDI in the United States.

**Disclosures:**

**Ruth Carrico, PhD, DNP, APR, CIC**, **Pfizer** (Consultant, Research Grant or Support, Speaker's Bureau)**Sanofi Pasteur** (Consultant, Grant/Research Support, Speaker's Bureau) **Fredrick J. Angulo, DVM, PhD**, **Pfizer, Inc.** (Employee) **Joann Zamparo, MPH**, **Pfizer, Inc.** (Employee) **Elisa Gonzalez, MS**, **Pfizer, Inc.** (Employee) **Kimbal D. Ford, PharmD**, **Pfizer, Inc.** (Employee) **Julio Ramirez, M.D., FACP**, **Pfizer, Inc.** (Scientific Research Study Investigator, Research Grant or Support, Speaker's Bureau)

